# Female Mice with HSD17B1 Inactivation Show Mild Hyperandrogenism without Notable Impact on Reproductive Function or Bone

**DOI:** 10.1210/endocr/bqaf167

**Published:** 2025-11-11

**Authors:** Arttu Junnila, Nataliia Petruk, Hanna Heikelä, Pekka Postila, Janne Hakkarainen, Guillermo Martinez-Nieto, Esperanza Uceda-Rodriguez, Francisco Ruiz-Pino, Manuel Tena-Sempere, Claes Ohlsson, Petra Sipilä, Terhi J Heino, Jorma Määttä, Matti Poutanen

**Affiliations:** Research Centre for Integrative Physiology and Pharmacology, Institute of Biomedicine, University of Turku, Turku FI-20520, Finland; Research Centre for Integrative Physiology and Pharmacology, Institute of Biomedicine, University of Turku, Turku FI-20520, Finland; Research Centre for Integrative Physiology and Pharmacology, Institute of Biomedicine, University of Turku, Turku FI-20520, Finland; Research Centre for Integrative Physiology and Pharmacology, Institute of Biomedicine, University of Turku, Turku FI-20520, Finland; Organon R&D Finland, Turku FI-20520, Finland; Research Centre for Integrative Physiology and Pharmacology, Institute of Biomedicine, University of Turku, Turku FI-20520, Finland; Turku Center for Disease Modeling, University of Turku, Turku FI-20520, Finland; Department of Cell Biology, Physiology and Immunology, University of Cordoba, and Instituto Maimónides de Investigación Biomédica de Córdoba, 14071 Córdoba, Spain; Department of Cell Biology, Physiology and Immunology, University of Cordoba, and Instituto Maimónides de Investigación Biomédica de Córdoba, 14071 Córdoba, Spain; Department of Cell Biology, Physiology and Immunology, University of Cordoba, and Instituto Maimónides de Investigación Biomédica de Córdoba, 14071 Córdoba, Spain; The Sahlgrenska Osteoporosis Centre, Department of Internal Medicine and Clinical Nutrition, Institute of Medicine, Sahlgrenska Academy, University of Gothenburg, 41345 Gothenburg, Sweden; Research Centre for Integrative Physiology and Pharmacology, Institute of Biomedicine, University of Turku, Turku FI-20520, Finland; Turku Center for Disease Modeling, University of Turku, Turku FI-20520, Finland; Research Centre for Integrative Physiology and Pharmacology, Institute of Biomedicine, University of Turku, Turku FI-20520, Finland; Research Centre for Integrative Physiology and Pharmacology, Institute of Biomedicine, University of Turku, Turku FI-20520, Finland; Turku Center for Disease Modeling, University of Turku, Turku FI-20520, Finland; Research Centre for Integrative Physiology and Pharmacology, Institute of Biomedicine, University of Turku, Turku FI-20520, Finland; The Sahlgrenska Osteoporosis Centre, Department of Internal Medicine and Clinical Nutrition, Institute of Medicine, Sahlgrenska Academy, University of Gothenburg, 41345 Gothenburg, Sweden

**Keywords:** HSD17B1, estrogens, steroidogenesis, female reproduction, bone health, hyperandrogenism

## Abstract

17β-hydroxysteroid dehydrogenase 1 (HSD17B1) is the primary enzyme responsible for the activation of estrone (E1) to estradiol (E2) in ovaries and extra-gonadal tissues of both humans and rodents. In the present study, molecular modeling identified the substitution of His222 in the human HSD17B1 enzyme with glycine in the mouse as the key determinant for the different steroid specificity between the species. Furthermore, Ser143Ala mutation at the active site of mouse HSD17B1 resulted in a total loss of E1 to E2 conversion by HSD17B1. This resulted in elevated intraovarian and circulating E1 concentrations in adult HSD17B1 Ser143Ala knock-in (HSD17B1-KI) females, but no changes in E2 concentrations were observed compared to the wild-type mice. Androstenedione and dihydrotestosterone were also elevated in the HSD17B1-KI ovaries, associated with elevated circulating LH. However, the effect of HSD17B1 inactivation on female reproductive development and function was mild, primarily resulting in a slight decrease in ovarian weight in older HSD17B1-KI mice, without notable effects on fertility. Expression of genes related to steroid biosynthesis, mitochondrial metabolism, and known markers of polycystic ovary syndrome was found to be upregulated in adult HSD17B1-KI ovaries. However, no alterations in the structure or function of extra-gonadal tissues were observed, and the uterus and bone phenotypes in the HSD17B1-KI females were unaffected. Our results demonstrate that the blockade of HSD17B1-dependent E2 synthesis is successfully compensated for in mouse in vivo, resulting in only a mild ovarian estrogen and androgen imbalance but no significant adverse effects on reproductive or bone health.

Several enzymes of the 17β-hydroxysteroid dehydrogenase (HSD17B) family have been shown to catalyze the conversion of 17-ketosteroids to 17β-hydroxysteroids or vice versa in vitro (1). Although we have previously demonstrated that the primary function of several of the HSD17B enzymes is outside of sex steroid synthesis, with only a minor role in estradiol (E2) synthesis in vivo (1), all the data support the assumption that HSD17B1 is a steroid biosynthetic enzyme involved in E2 synthesis both in experimental rodent models and in humans. However, the mouse HSD17B1 has been shown to catalyze the conversion of androstenedione (A4) to testosterone (T) and E1 (estrone) to E2 with similar efficiencies, whereas the human enzyme is more specific for E2 synthesis (2, 3). HSD17B1 highly prefers nicotinamide adenine dinucleotide phosphate (NADPH) as a cofactor, which primarily determines the E1-to-E2 reaction direction in vivo (4).

In both humans and mice, HSD17B1 is primarily expressed in ovarian granulosa cells. It is also highly expressed in human placental syncytiotrophoblasts but not in the mouse placenta (4, 6, 7), which, unlike humans, does not produce a significant amount of E2. Granulosa cells produce estrogens from theca cell-produced androgens (A4 and T) with a coordinated expression of P450 aromatase and HSD17B1 (5). The expression of HSD17B1 in rodent ovarian follicles is induced at an early stage and remains high throughout follicular development but is dramatically downregulated in luteinization (6).

Furthermore, a low level of HSD17B1 expression has been consistently observed in extragonadal reproductive tissues in women, such as normal and malignant endometrium and breast tissue, indicating that the enzyme may have a role in regulating local E2 concentrations in estrogen-responsive tissues as well (7-9). Thereby, the enzyme is also considered to be involved in the pathophysiology of estrogen-driven diseases, such as endometriosis and endometrial and breast cancer (10, 11). Accordingly, the inhibition of HSD17B1 to lower the target tissue E2 concentration has been considered as a potential treatment option for estrogen-dependent diseases. Several inhibitors have already been evaluated in preclinical models, with 1 phase 1 clinical study conducted (11-13). Therefore, detailed knowledge about the effect of chronic HSD17B1 inhibition on ovarian steroid synthesis, female reproductive function, and other estrogen target organs is required to predict any potential unfavorable effects associated with pharmacological inhibition of the enzyme.

We have previously generated an HSD17B1 knockout mouse line in which the entire 2-kb gene was replaced with a lacZ-expressing targeting vector (14). Later on, it was recognized that the gene replacement resulted in a dramatic downregulation of the expression of an upstream gene, *Naglu*, leading to a metabolic phenotype unrelated to the HSD17B1 function (15). We have, furthermore, observed that the reproductive disruption in male HSD17B1 knockout mice with the lacZ insertion was not fully replicated in the mouse line with an inactivating point mutation of HSD17B1 (16, 17). Therefore, the present study aimed to investigate the effects of a point mutation at the active site of HSD17B1 in female mice. The particular substitution of Ser143, part of the active site motif shared across HSD17B enzymes (18), with alanine has previously been shown to result in nearly complete inactivation of the enzyme in vitro (2, 17). Additionally, molecular modelling was used to further clarify the underlying factors at the structure level contributing to the observed differences in substrate specificity between human and mouse HSD17B1 enzymes.

## Materials and Methods

### Molecular Modeling

The human E1-HSD17B1 complex was modeled based on the X-ray crystal structure of the E2-HSD17B1-NADP^+^ complex, acquired from the Protein Data Bank (PDB; 1A27; https://www.rcsb.org/; accessed August 2, 2024) (19). First, the rotamer of Glu283 sidechain was adjusted in BODIL (20) to better facilitate direct hydrogen-bonding (H-bonding) with the substrate's C3-hydroxyl. Second, the system was protonated at pH 7.4, C17 position H-bonding and protonation of the bound E2 was edited to match E1 (hydroxyl to carbonyl), and ligand-protein binding minimization was performed using MAESTRO (Schrödinger Release 2023-3: Maestro, Schrödinger, LLC, New York, NY, USA, 2022).

The PDB entry 1A27 was directly used via ligand-protein binding minimization as a template for generating the optimized human E2-HSD17B1 complex. Similarly, the human A4-HSD17B1 complex was modeled directly based on the X-ray crystal structure of the A4-HSD17B1 complex (PDB: 1QYW; accessed August 2, 2024) (21). Lastly, the reaction product T-HSD17B1 complex was also modeled via ligand-protein binding minimization directly based on human T-HSD17B1 complex X-ray crystal structure (PDB: 1JTV; accessed August 2, 2024) (22). No large-scale adjustments occurred during the optimization of the crystal structure.

**Figure 1.**
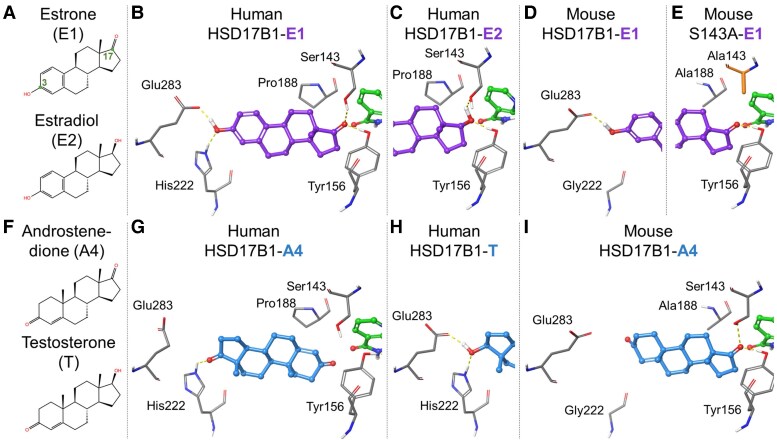
Human and mouse HSD17B1 substrate binding modes. (A) 2D representations of E1 (substrate) and E2 (product). (B) The minimized human E1-HSD17B1 complex indicates that the C3-hydroxyl can form a double H-bond with both Glu283 and His222, and the C17-keto group can also optimally H-bond with Ser143 and Tyr156. (C) After the E1-to-E2 reaction, the C17-hydroxyl positioning of E2 at the catalytic end is compromised due to the proximity of multiple H-bond donors. (D) In mouse HSD17B1, Gly222 is present at the noncatalytic end of the enzyme, instead of the histidine, but the carboxylate group of Glu283 can H-bond directly with the C3-hydroxyl of E1. (E) Mutating Ser143 to alanine compromises or abolishes the C17-keto coordination needed for acquiring the proton from NAD (P)H via the phenol ring of Tyr156. (F) 2D representations of A4 (substrate) and T (product). (G) With the minimized human A4-HSD17B1 complex, the C17-keto group H-bonds with the epsilon protonated His222, preferring a binding mode that is reverse and nonproductive for the reaction. (H) Likewise, in the energy-minimized human T-HSD17B1 complex, the C17-hydroxyl also acquires the double H-bond arrangement with Glu283 and His222. (I) In the modeled mouse A4-HSD17B1 complex, the reverse binding mode is not favored as there are no suitable H-bond donors present at the noncatalytic end. The protein residues are shown as stick models with grey backbones. The ligands and cofactors (NADP+) are shown as ball-and-stick models with magenta/blue or green backbone, respectively. Human and mouse HSD17B1 residue numbering is taken from the UniProt entries DHB1_HUMAN (P14061) and DHB1_MOUSE (P51656). Abbreviations: A4, androstenedione; E1, estrone; E2, estradiol; HSD17B1, 17β-hydroxysteroid dehydrogenase 1; NADP, nicotinamide adenine dinucleotide phosphate; T, testosterone.

The mouse E1-HSD17B1 and A4-HSD17B1 complexes were modeled by aligning the mouse HSD17B1 AlphaFold Database model (AF-P51656-F1-model_v4; https://alphafold.ebi.ac.uk/; accessed August 2, 2024) (23) against the human PDB entries 1A27 and 1QYW, respectively, using the protein backbone C^α^ atoms with VERTAA in BODIL (20). The bond information of the ligands was corrected before extracting and merging them with the mouse AlphaFold 2 model. The protonation was set to match pH 7.4, and ligand-protein binding minimization was applied in MAESTRO. The mouse HSD17B1-S143A mutant was generated based on the mouse E1-HSD17B1 complex without applying minimization. For reference, the cofactor NADP^+^ was extracted from the PDB entry 1A27 and added to all the modeled HSD17B1 systems. Figures 1 and S1 were generated using MAESTRO.

### Enzymatic Activity

The enzymatic activity of mouse HSD17B1 with Ser143Ala point mutation was analyzed in cell culture using E1 as a substrate. Briefly, the point mutation was introduced to a wild-type (WT) mouse *Hsd17b1* expression plasmid (Origene) with a Q5 Site Directed Mutagenesis Kit (NE Biolabs) according to manufacturer instructions, with the following primers: forward 5′-GTGACCGCGGCAGTGGGAGGCTT-3′, reverse 5′-CAGCACACGCCCAGAGTGGC-3′. The mutant and WT plasmids were transformed into the *E. coli* strain provided with the kit, and the transformed cells were plated on kanamycin selection plates overnight. The colonies were then picked and maxi-preps were prepared, cultured overnight, and purified with the NucleoBond Xtra Maxi Plus plasmid purification kit (Macherey-Nagel). The purified plasmids expressing the Ser143Ala mutant or WT mouse HSD17B1 were transfected into cultured MCF-7 cells, and the HSD17B activity of the cells was determined by adding tritium-labeled E1 to the cells in medium and terminating the reaction at 1 hour, 3 hours, and 7 hours later. The conversion of E1 to E2 was calculated by analyzing the substrate-to-product ratio, separated by high-performance liquid chromatography connected to a beta-counter.

### Mice Used in the Study

The mouse line with the *Hsd17b1* Ser143Ala point mutation (HSD17B1-KI) was generated using the CRISPR/Cas9 gene editing system as previously described (17). The Ser143Ala point mutation in the germ line was previously confirmed by DNA sequencing. The studies used homozygous mutant female mice, as well as WT female mice as controls, obtained by breeding heterozygous mutant males and females. All animal work was conducted at the Central Animal Laboratory at the University of Turku, Finland, under the animal license numbers 41729/2019 and 23322/2023, granted by the Project Authorisation Board (formerly the Animal Experiment Board) in Finland. The handling of the mice fully meets the requirements as defined in the National Institutes of Health (Bethesda, MD, USA) guidelines for the care and use of laboratory animals.

Mice were housed in individually ventilated cages (Techniplast, Buguggiate, Italy) with approximately 70 air changes per hour. A constant temperature of 21 ± 3 °C and humidity of 55 ± 15% were maintained, along with a 12-hour light-dark cycle, with light changes at 7 Am and 7 Pm. The mice were housed with their littermates, 1 to 6 mice per cage. Autoclaved aspen chips were used as bedding mooaterial (Tapvei Ltd, Harjumaa, Estonia), and each cage was supplied with a plastic nest box and paper tissue as nesting material. Soy-free pellets (RM3, Special Diets Services, Essex, UK) and water were available ad libitum.

A cohort of 10 WT and 11 HSD17B1-KI females was sacrificed at the age of 8 weeks. Another cohort of 10 WT and 10 HSD17B1-KI females was sacrificed at the age of 6 months. The mice were euthanized using CO_2_-asphyxiation followed by cervical dislocation. Blood was collected via heart puncture, allowed to coagulate at room temperature (RT), and then centrifuged at 3000×g for 15 minutes. Subsequently, serum was collected and stored at −20 °C for further analysis. Body weight and macroscopic anatomy of reproductive organs (ovary and uterus), adipose tissue (gonadal fat), spleen, liver, and pituitary were recorded. Dissected tissues were either snap-frozen in liquid nitrogen and stored at −80 °C or fixed in 10% neutral buffered formalin at RT for 24 hours (unless otherwise stated) and embedded in paraffin blocks (see later discussion). Femurs and tibia were dissected and cleaned of surrounding soft tissues, fixed and subjected to micro-computed tomography (µCT) analyses prior to decalcification and embedding in paraffin blocks (see later discussion).

### Determining Puberty Onset and Estrous Cycle

Puberty onset was determined by observing the vaginal opening in 13 WT and 13 HSD17B1-KI females for 14 consecutive days, starting at postnatal day 21. The estrous cycle regularity was monitored in 8-week-old females for 21 consecutive days. Vaginal cytology samples were collected from 10 WT and 10 HSD17B1-KI females every morning by vaginal lavage with approximately 100 µL of PBS. The sample was then transferred to a microscope slide and allowed to dry at RT. After drying, the samples were briefly fixed in 96% and 100% ethanol and rinsed in distilled water. The samples were stained with Mayer's hematoxylin solution (Histolab Products, Askim, Sweden) for 1 minute and rinsed in tap water. Before mounting with Pertex (Histolab Products), the samples were dehydrated in a series of ethanol and xylene.

Slides were then scanned with Pannoramic P 1000 slide scanner (3DHISTECH Ltd., Budapest, Hungary), and the estrus cycle phase was defined based on the presence or proportions of the cell types using a computerized, deep learning-based method created in the Aiforia (RRID: SCR_022739) Create (version 5.3, Aiforia Technologies, Helsinki, Finland), as previously described (24).

### Studying Fertility and Mating Behavior

Sixteen WT and 15 HSD17B1-KI females were mated with WT males at the age of 8 weeks. The litter size, sex ratio, and mean body weight of the pups were recorded for 2 consecutive litters from each female. For embryo analysis, 14 WT and 14 HSD17B1-KI females were mated with WT males at the age of 4 months, then sacrificed at 13.5 days post coitum. The uteri were dissected, and the number of dead and alive embryos, the weight of the ovaries, and the mean weight of placentas were recorded.

### Superovulation

Female mice, 3 to 4 months of age, were treated with 5 IU of pregnant mare serum gonadotropin (PMSG), and 48 hours later, 1 group of mice (WT, n = 7; HSD17B1-KI, n = 7) was sacrificed. The remaining PMSG-treated mice (WT, n = 7; HSD17B1-KI, n = 7) received 5 IU of human chorionic gonadotropin, and the animals were sacrificed the following morning. The released oocytes were collected from the oviducts and counted. Both compounds were administered intraperitoneally.

### Steroid Measurements

The concentrations of serum A4, T, dihydrotestosterone (DHT), progesterone, E1, and E2 were analyzed by a validated gas chromatography-tandem mass spectrometry, with the quantification limits of 12 pg/mL, 8 pg/mL, 2.5 pg/mL, 74 pg/mL, 0.5 and 0.5 pg/mL, respectively. The method was also used to measure intratissue steroid concentrations after homogenizing the tissues in sterile deionized water (1:10 w/v) using an Ultra-Turrax homogenizer (IKA-Werke, Staufen im Breisgau, Germany) as previously described (25).

### LH Measurements

Serum samples were assayed using an ultra-sensitive LH ELISA on a 96-well high-affinity binding microplate (Costar Assay Plate, Corning) as previously described (26). 50 ng/well of monoclonal anti-bovine LHβ subunit antibody (Pablo Ross; UC Davis Cat# 518B7; RRID: AB_2756886) was used as the capture antibody, and 250 ng/well of mouse LH antibody (Medix Biochemica Cat# 100588; RRID: AB_2784503), which was biotinylated using an EZ-Link NHS-PEG4 Biotinylation Kit (Thermo Fisher Scientific, Cat# PI21455) was used as the detection antibody. A serial dilution of a LH reference, rLH-RP3 (AFP5306A; National Hormone and Pituitary Program, CA, USA), in PBS-Tween was used to produce a standard curve. Detection was achieved with poly-horseradish peroxidase conjugate streptavidin (Thermo Fisher Scientific, Cat# N200), diluted 1:20 000, followed by 100 μL/well of o-phenylenediamine (Sigma-Aldrich Corp., St. Louis, MO, USA) diluted in citrate buffer (pH 6) with 0.1% H2O2 and stopped with HCl (3 M). Absorbance was measured (Bio-Rad iMARK) at 490 and 665 nm for background subtraction. The assay sensitivity was 0.002 ng/mL.

### Histology

Soft tissues were fixed in 10% neutral buffered formalin (FF-Chemicals, Haukipudas, Finland) for approximately 24 hours at RT, dehydrated and embedded in paraffin, and 5-µm-thick sections were prepared. For analysis, the sections were deparaffinized and rehydrated, stained with hematoxylin and eosin, and examined via light microscopy.

### Gene Expression Analysis and RNA Sequencing

Total RNA was extracted from the ovaries of 2-month-old (WT n = 5; HSD17B1-KI, n = 5), 4-month-old (pregnant cohort; WT n = 9; HSD17B1-KI n = 11), and 6-month-old (WT n = 8; HSD17B1-KI, n = 6) female mice with Trisure (Bioline, London, UK) following the manufacturer's instructions. The RNA integrity in the samples was confirmed with a NanoDrop ND-1000 spectrophotometer (Thermo Fisher Scientific). One µg of RNA was treated with a DNase Amplification Grade Kit (Thermo Fisher Scientific) and used for cDNA synthesis (SensiFast, Bioline). The cDNA was used to quantify gene expression by quantitative PCR (CFX96 Real-Time PCR detection system; Bio-Rad, Hercules, CA, USA) with the DyNAmo Flash SYBR Green qPCR Kit (Thermo Fisher Scientific). The primers used are shown in Table 1. Data were normalized to the expression of the housekeeping gene (β-actin), and expression was calculated using the ΔΔCt method. To account for gene expression changes during the estrous cycle, only samples obtained at the estrus phase of the cycle were analyzed.

**Table 1.** qPCR primers used for mRNA expression analysis

Gene	Forward primer sequence	Reverse primer sequence	Gene accession number
*Actb*	CGTGGGCCGCCCTAGGCACCA	TTGGCCTTAGGGTTCAGGGGG	NM_007393.5
*Cyp17a1*	CAAGCCAAGATGAATGCAGA	AGGATTGTGCACCAGGAAAG	NM_007809.3
*Hsd17b1*	TTGTTTGGGCCGCTAGAAG	CACCCACAGCGTTCAATTCA	NM_010475.2
*Star*	CAGGGAGAGGTGGCTATGCA	CCGTGTCTTTTCCAATCCTCTG	NM_011485.5

Full names of genes: beta-actin; cytochrome p450, family 17, subfamily A, polypeptide 1; hydroxysteroid dehydrogenase 1; steroidogenic acute regulatory protein.

Abbreviation: qPCR, quantitative PCR.

The RNA extracted from the ovaries of 6-month-old WT and HSD17B1-KI females in estrus was further used for global transcriptomic analysis by RNA sequencing. The quality of the RNA samples was confirmed by Bioanalyzer (Agilent, Santa Clara, CA, USA). Library preparation and sequencing were performed by CeGaT GmbH. (Tuebingen, Germany). Library preparation was performed using the Illumina TruSeq Stranded mRNA kit, and sequencing was conducted with the Illumina NovaSeq 6000 system.

For the analysis of the sequencing results, demultiplexing of the sequencing reads was performed with Illumina bcl2fastq (version 2.20). Adapters were trimmed using Skewer (version 0.2.2) (27). Trimmed raw reads were aligned to mm10 using STAR (version 2.7.3) (28). Differential expression between the genotypes was analyzed using DESeq2 (version 1.24.0) (31) in R (version 4.0.4), employing a negative binomial generalized linear model to test for differential expression based on gene counts. The differentially expressed genes were further visualized with the STRING protein interaction network database (version 12.0) and functional enrichment analysis (29). The results were clustered with Markov cluster algorithm, with an inflation factor of 2.5.

### Bone Measurements, µCT Analysis, and Histology

Femur and tibia lengths, as well as femoral diameter in both sagittal and coronal planes, were measured using a caliper from bones collected from 2-month-old (WT n = 10, HSD17B1-KI n = 11) and 6-month-old (WT n = 10, HSD17B1-KI n = 10) female mice. The bones were subsequently used for histomorphometric and histological analysis.

Quantitative analysis of the femurs before decalcification and embedding was performed using a Skyscan 1272 X-ray computer tomography scanner (Bruker, Kontich, Belgium). Cross-sectional image stacks were reconstituted with NRecon 1.6.9.16 software (Bruker Skyscan). Morphometric parameters, including tissue volume (mm^3^), bone volume (mm^3^), and bone volume/tissue volume (percent), were analyzed using CTan (version 1.9.32) software from Bruker Skyscan. The parameters applied for scanning were as follows: X-ray tube voltage, 70 kV; tube current, 148 μA; X-ray filtration with a 0.5 mm aluminum filter; and 5 μm resolution. The trabecular bone morphometric region of interest was defined at the metaphysis of the femur, starting 50 layers (250 μm) below the bottom of the growth plate as indicated by the appearance of woven bone at the middle of the cross-sectional image and extending 150 layers for 2-month-old mice and 100 layers for 6-month-old mice. Cortical bone parameters were measured from a 50-layer-thick disc 900 layers below the same anatomical marker used for the trabecular bone analysis. Model images of the femoral heads were generated with CTVox (version 3.3.0) image rendering program (Bruker Skyscan).

For bone histology, tibia were fixed in 10% neutral buffered formalin, decalcified for 2 weeks in 10% EDTA (pH 7.2) with slow agitation and daily liquid change, and embedded in paraffin. Sections were then cut as previously described (30). Osteoclasts were stained for tartrate-resistant acid phosphatase (TRAcP) (Merck, Germany), and the number of osteoclasts was manually counted in the area below the cartilagenous growth plate using Fiji-ImageJ (version 1.52p) software (31). The region of interest expanded throughout the histological bone section.

### Human Osteoclast and Osteoblast Cultures

CD14-positive cells isolated from peripheral blood samples drawn from 5 premenopausal women (University of Turku Ethics Committee Statement #59/2019) were seeded in 48-well plates (1 × 10^5^ cells/well) in α-MEM media supplemented with 10% charcoal-stripped fetal bovine serum (iFBS; Thermo Fisher Scientific, A3382101), 1% penicillin/streptomycin (Gibco), and 1% Glutamax (Gibco) and left to adhere for 24 hours. For osteoclast differentiation, the medium was changed to complete α-MEM supplemented with 10% iFBS, 10 ng/mL macrophage colony-stimulatory factor (R&D Systems, 216-MC-025), and 20 ng/mL receptor activator of nuclear factor kappa-B ligand (PeproTech, 310-01). The HSD17B1 inhibitor (iHSD17B1) used in the experiments was compound 21 presented in a previous publication (32). Cells were cultured for 10 days in a medium containing either vehicle (dimethyl sulfoxide), iHSD17B1 (1 µM), E1 (1 nM), E2 (1 nM), iHSD17B1 + E1, or iHSD17B1 + E2 (n = 5 per group). Half of the medium was replenished every 3 to 4 days.

Human primary osteoblast (hOB) cells from a healthy premenopausal female donor were purchased from PromoCell (C-12720, 483Z004.2) and expanded in osteoblast culture medium (PromoCell, C-27015) supplemented with 10% iFBS, 1% penicillin/streptomycin, and 1% Glutamax (Gibco). Cells at passages 4 to 7 were used for the experiments. As a second model, human primary mesenchymal stem cells (MSCs) from a healthy premenopausal female were purchased from PromoCell (C-12974, 439Z037.1) and maintained in Mesenchymal Stem Cell Growth Medium 2 (PromoCell, C-28009). Both hOB cells and MSCs were seeded at a density of 10 000 cells/cm^2^ into 48-well cell culture plates and cultured in osteoblast culture media (PromoCell, C-27015) supplemented with 10% iFBS, 50 µg/mL L-ascorbic acid, and 10 mM sodium β-glycerophosphate for 14 and 21 days in a medium containing either vehicle (dimethyl sulfoxide), iHSD17B1 (1 µM), E1 (1 nM), E2 (1 nM), iHSD17B1 + E1, or iHSD17B1 + E2. Half of the medium was replenished every 3 to 4 days.

For analyzing the HSD17B activity (conversion of E1 to E2 and E2 to E1) in osteoclast, hOB, and MSC cultures, the last medium replenishment with a full volume of fresh medium containing the substances was performed on day 9 for osteoclasts and on day 13 or 20 for hOBs and MSCs. After 24 hours of incubation with cells, the media were collected for steroid analysis. Media without cells was included as a control in the analysis (n = 2 for osteoclast and n = 4 for osteoblast experiments).

### Statistical Analyses

Statistical analyses were carried out using GraphPad Prism (versions 9 and 10) software (GraphPad Software, Inc., La Jolla, CA, USA). Outliers were identified using the ROUT method with a coefficient of Q = 1. Data normality was tested with the Shapiro-Wilk test, and the statistical test was selected based on data normality. When comparing 2 groups at a single time point, the statistical significance was determined using the unpaired *t*-test or the nonparametric Mann-Whitney test. One-way ANOVA was used for comparisons of several groups at a single time point, with correction for multiple comparisons. When comparing groups across various time points, a 2-way ANOVA was used. The statistical significance was set at a *P*-value of less than .05. Data are represented as individual values and means or as box plots that present median values, upper and lower quartiles, as well as minimum and maximum scores.

## Results

### The Catalytic Triad of HSD17B1 Is Broken by S143A Mutation

A characteristic of both human and mouse HSD17B1 is the catalytic triad Ser-Tyr-Lys, which facilitates the reduction of E1 to E2 at the C17 position of the steroidal ring (18). More specifically, the C17-keto group of E1 must be positioned toward the Tyr-Lys-Ser of the catalytic triad prior to the reaction (Fig. 1A and 1B), which is achieved through the ability of the C3-hydroxyl group of E1 to form a double H-bond with the side chains of Glu283 and His222 at the noncatalytic end of the active site of the human enzyme. The highly coordinated C3-hydroxyl arrangement was modeled using the X-ray crystal structure of the existing E2-HSD17B1-NADP^+^ complex (Fig. 1C), and the positioning closely matched that observed in the crystal structure of the equilin-HSD17B1 complex (PDB: 1EQU) (33). Although mouse HSD17B1 also reduces E1 to E2, the presence of glycine instead of histidine at position 222 weakens the binding for the substrate at its C3-end (Fig. 1D), which is thus expected to reduce the catalytic rate as well.

The phenol of the tyrosine side chain at the active site has been suggested to act as the primary proton source for the oxidoreductive transfer of a hydride, during which the cofactor NAD (P)H is oxidized to NAD (P)^+^. The position of the lysine is expected to lower the pKa value of tyrosine, allowing for efficient proton detachment during the reaction. Notably, the positively charged lysine is bound to the cofactor in HSD17B1 X-ray crystal structures containing NAD (P)+, and thus, a notable sidechain rotation or mobility would be required to facilitate its suggested function at the active site. In this scheme, the role of Ser143 is to facilitate the optimal placement of the C17-keto group in relation to the serine, lysine, and NAD (P)H. Accordingly, by mutating Ser143 to alanine in mouse (as well as human) HSD17B1, the reduction reaction is expected to be effectively prevented (Fig. 1E), as also experimentally evidenced in the present study.

### Differences in Active Site Binding Lead to Distinct Substrate Preferences between Mouse and Human HSD17B1

An HSD17B1 active site can also accept androgens as substrates (Fig. 1F), and the X-ray crystal structure of human A4-HSD17B1 complex (Fig. 1G) indicates that the substrate's C17-keto group can form a H-bond with the epsilon-protonated imidazole side chain of His222. This causes a tendency for A4 to be locked into the reverse binding mode, preventing the reductive reaction from taking place at C17 of the substrate. The same reverse binding mode, in which the C17-hydroxyl is H-bonded to both the His222 and Glu283 side chains, is also readily available for T, being the expected product of the reaction (Fig. 1H). However, because His222 is replaced by glycine in mouse HSD17B1, the A4-to-T reaction can still occur, albeit slowly, due to the absence of H-bonding with noncatalytic end residues (Fig. 1I). Accordingly, the A4 has the potential to bind at the mouse active site with its C17-keto group correctly facing the catalytic triad residues for achieving the reduction reaction.

The differences of 3 other residues at the HSD17B1 active site between human and mouse enzymes may also affect the binding of E1 and A4. First, the replacement of Pro180 with alanine in the mouse enzyme counterpart could affect the overall steroid ring alignment (Fig. 1G and 1I). Second, although positioned relatively far from the catalytic residues, the replacement of Asn153 with a longer-reaching histidine could potentially affect ligand binding at the active site (not shown). Lastly, swapping Ser223 with tyrosine places a hydroxyl group within a striking distance of the substrate's C17-keto group (not shown).

A more comprehensive 2D visualization of the interactions and the residues surrounding the binding pockets of the human and mouse HSD17B1 enzymes, and of the mutant mouse HSD17B1, is presented in Fig. S1 (34). The amino acid sequence differences at the active site between human and mouse HSD18B1 are highlighted in Fig. S2 (34).

### The Lack of HSD17B1 Activity in Female Mice is Compensated for by Altered Ovarian Steroidogenesis

**Figure 2.**
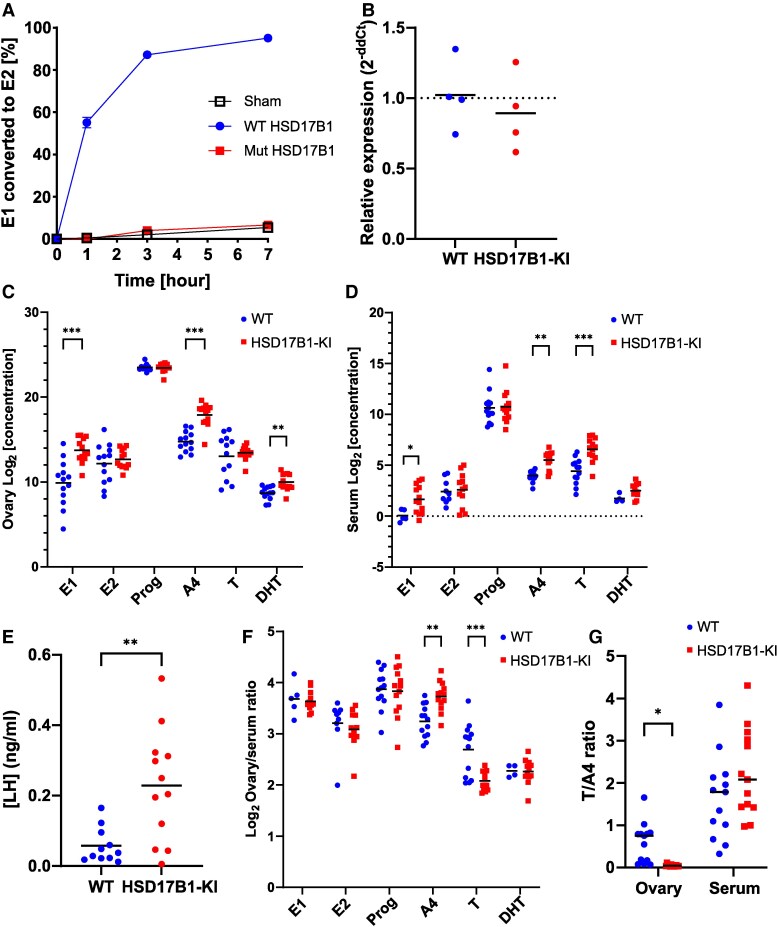
HSD17B1 inactivating mutation affected ovarian steroidogenesis. (A) Conversion of E1 to E2 in MCF-7 cells transfected with Ser143Ala mutant (Mut) HSD17B1 is greatly impaired in comparison with cells transfected with WT HSD17B1, being equal to nontransfected cells (Sham). (B) No change was observed in *Hsd17b1* mRNA expression in adult mouse ovaries analyzed by quantitative PCR (n = 4). (C) Intraovarian E1, A4, and DHT concentrations were increased in 6-month-old females, but other steroids were unchanged (n = 13). (D) Serum E1, A4, and T concentrations were also increased in the same animals. (E) Serum LH concentrations were increased in 6- to 7-month-old females (WT n = 11, HSD17B1-KI n = 12). (F) Ovary/serum steroid concentration ratio of A4 was increased, and T decreased in the HSD17B1-KI females. (G) The ratio of T to A4 was decreased in the HSD17B1-KI ovary but unchanged in serum. In (A), data are presented as means and SD; in (B-F), data are presented as individual values, with lines indicating means; and in (C, D, F), data were log_2_-transformed prior to analysis. *=*P*  *<* .05, **=*P* < .01, ***=*P* < .001. Abbreviations: A4, androstenedione; DHT, dihydrotestosterone; E1, estrone; E2, estradiol; HSD17B1, 17β-hydroxysteroid dehydrogenase 1; HSD17B1-KI, 17β-hydroxysteroid dehydrogenase 1 Ser143Ala knock-in; T, testosterone; WT, wild-type.

We have previously described the generation of the mouse model and the genotyping strategy, with confirmed germline transmission of the Ser143Ala mutation (17). As expected, the mutation resulted in the complete loss of the enzyme activity to convert E1 to E2 in transfected cells (Fig. 2A), while the point mutation did not affect *Hsd17b1* mRNA expression in the adult ovary (Fig. 2B).

The concentration of E1 was 6-fold higher (*P* < .001) in the ovaries of the 6- to 7-month-old female HSD17B1-KI mice when compared to WT mice, accompanied by a 3-fold higher A4 (*P* < .001) and 2.5-fold higher DHT (*P* < .01) concentration (Fig. 2C). However, no differences were observed in the ovarian concentrations of E2, progesterone, or T. These data, thus, indicate that the lack of HSD17B1 activity in the ovaries of the HSD17B1-KI females is efficiently compensated for by the increased local concentrations of A4 and E1, providing the precursor steroids for E2 production via the activities of other, less active HSD17B enzymes and P450 aromatase.

A 4-fold higher E1 concentration (*P* < .05) was also observed in the serum of HSD17B1-KI females (Fig. 2D) compared to WT females, with a 3-fold higher A4 (*P* < .01). Interestingly, serum T was also 4-fold higher (*P* < .001) in HSD17B1-KI, and DHT showed an increasing trend, whereas E2 and progesterone remained unchanged also in circulation (Fig. 2D). The altered steroid profile in HSD17B1-KI females was accompanied by a 4-fold higher serum LH (*P* < .01, Fig. 2E) compared to WT females. No difference was observed in the ovary/serum ratio of E1 or E2 concentration. However, the ovary/serum ratio of A4 was higher in HSD17B1-KI (*P* < .01), and the ratio of T was conversely lower (*P* < .001, Fig. 2F). The ratio of T to A4 was significantly reduced in HSD17B1-KI ovaries, likely indicating efficient further aromatization of excess A4 preferentially to E1 and T to E2, whereas in serum the ratio was similar to WT controls, reflecting a tendency of excess A4 to be converted to T peripherally (Fig. 2G).

### The Inactivation of HSD17B1 Did Not Significantly Disrupt the Reproductive Phenotype of Female Mice

**Figure 3.**
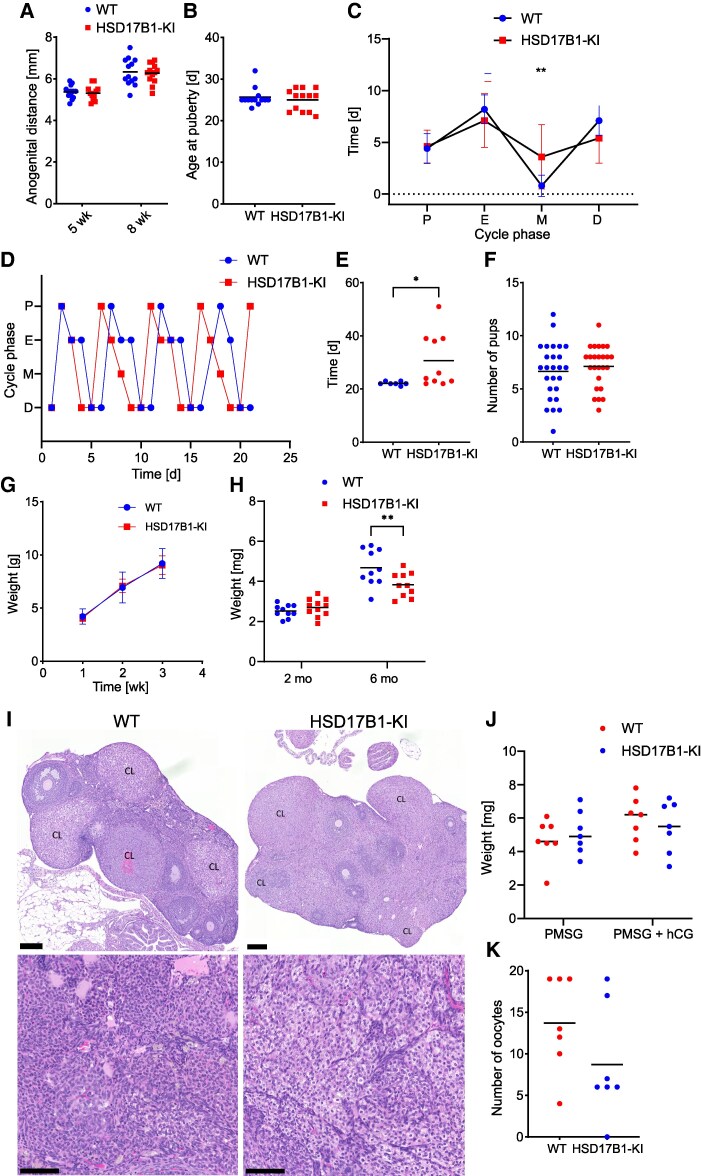
Female development and reproduction were not overtly disturbed by HSD17B1 inactivation. (A) Anogenital distance did not differ between WT (n = 13) and HSD17B1-KI (n = 13) females at the ages of 5 and 8 weeks, nor did (B) the timing of the onset of puberty. (C) Although the HSD17B1-KI females cycled mostly normally, a small increase was seen in the number of total days spent in the metestrus stage over the 4 cycles followed. (D) Representative diagrams of estrous cycle progression. (E) Mean time between spontaneous delivery of litters was delayed in HSD17B1-KI females in constant breeding for 2 months (n = 10). (F) Number of pups in litters after spontaneous delivery did not differ between the groups, nor did (G) weights of pups over the first 3 weeks after birth. (H) Ovary weights did not differ between WT and HSD17B1-KI females at 2 months of age (WT n = 10, HSD17B1-KI n = 11) but were decreased in HSD17B1-KI at 6 months of age (n = 10). (I) Representative ovarian histology, showing current and regressing corpora lutea and the prominent interstitial cells in HSD17B1-KI in comparison to WT histology. (J) Weights of ovaries did not differ between groups (n = 7) after stimulation with PMSG and hCG, nor did (K) the number of released oocytes after superovulation. In (A, B, E, F, H, J, K), data are presented as individual values, with lines indicating the means; in (C, G), data are presented as means and SD. In (I), scale bars in wider field images indicate 200 µm and in closer images 100 µm. *P  *<* .05, **P < .01. Abbreviations: hCG, human chorionic gonadotropin; HSD17B1, 17β-hydroxysteroid dehydrogenase 1; HSD17B1-KI, 17β-hydroxysteroid dehydrogenase 1 Ser143Ala knock-in; PMSG, pregnant mare serum gonadotropin; WT, wild-type.

The external appearance of the female mice was not affected by the HSD17B1 inactivation, and the mutant mice exhibited normal genitalia and nipple development. No differences were observed in the anogenital distances between WT and HSD17B1-KI females at 5 weeks or 8 weeks of age (Fig. 3A). The onset of puberty in the offspring of the HSD17B1-KI females was also unaffected, occurring at an average age of 25 days as monitored by vaginal opening (Fig. 3B). The HSD17B1-KI females progressed through the estrous cycle mostly normally but appeared to spend a longer time in the metestrus phase of the cycle (Fig. 3C and 3D). In a breeding test, the interval between litters was observed to be delayed for some of the HSD17B1-KI females. In contrast, it was very regular in the WT group (Fig. 3E). However, the litter sizes or the average weights of the pups over the first postnatal weeks did not differ between the genotypes (Fig. 3F and 3G). When pregnant dams were sacrificed at 13.5 days post coitum, no difference was observed in the number of living or deceased embryos or the number or weight of placentas, as presented in Fig. S3A to S3C (34).

The weight of the ovaries did not differ between the genotypes at the age of 2 months. However, at 6 months, the weight of the HSD17B1-KI ovaries was 20% smaller than that of the WT ovaries (*P* < .01) (Fig. 3H). A similar 30% difference between the genotypes was also observed in the ovary weights of 5-month-old pregnant dams sacrificed at 13.5 days post coitum (Fig. S3D) (34). Despite the difference in ovary weight, no major changes were identified in ovarian histology at the ages of 2 or 6 months (Fig. 3I). Based on histological analysis, both WT and HSD17B1-KI females presented with corpora lutea of the current cycle, as well as regressing corpora lutea. This, together with the data from the vaginal smears, proved regular cycling of HSD17B1-KI females. However, in some HSD17B1-KI ovaries from the 6-month-old mice, interstitial cell hypertrophy and hyperplasia were identified, exemplified by large areas of granular cells (Fig. 3I).

To further determine whether the difference in ovary size at old age could indicate premature ovarian failure or insufficiency, we super-ovulated 3- to 4-month-old female mice with either PMSG or a combination of PMSG and human chorionic gonadotropin. No difference was observed between the genotypes in the weights of ovaries following the treatments (Fig. 3J), nor was there a difference in the number of released oocytes (Fig. 3K), further indicating a normal ovulatory response. Female HSD17B1-KI mice exhibited a slightly lower body weight at the age of 2 months (Table 2); however, this difference was no longer evident at the age of 6 months (Table 3). Furthermore, no differences were observed in the weights of any of the analyzed tissues, including the adrenals, spleen, liver, or gonadal white adipose tissue, or the wet weight of the uterus, either in total or when accounting for the estrous cycle stage (Tables 2 and 3).

**Table 2.** Body and tissue weights of 2-month-old WT and HSD17B1-KI females

	Body weight (g)	Uterus wet weight*^a^* (mg)	Adrenal weight (mg)	WAT weight (mg)	Spleen weight (mg)	Liver weight (mg)
**WT**	19.1 ± 0.65	119.4 ± 24.43	4.31 ± 0.78	247.2 ± 62.61	68.4 ± 11.71	903.1 ± 114.73
**HSD17B1-KI**	17.7 ± 1.55	83.6 ± 37.93	3.9 ± 0.64	201.8 ± 67.52	60.4 ± 9.41	831.5 ± 111.30
***P*-value**	***.023***	.144	.226	.146	.117	.184

Values are presented as means ± SD. Significant (< 0.05) *P*-values are indicated in bolded italics.

Abbreviations: HSD17B1-KI, 17β-hydroxysteroid dehydrogenase 1 Ser143Ala knock-in; WAT, white adipose tissue; WT, wild-type.

^*a*^Determined from samples in the proestrus or estrus phase.

**Table 3.** Body and tissue weights of 6-month-old WT and HSD17B1-KI females

	Body weight (g)	Uterus wet weight*^a^* (mg)	Adrenal weight (mg)	WAT weight (mg)	Spleen weight (mg)	Liver weight (mg)
**WT**	28.0 ± 3.95	101.8 ± 24.35	7.6 ± 0.89	1206.1 ± 465.57	80.3 ± 9.46	1118.7 ± 135.31
**HSD17B1-KI**	29.1 ± 4.20	82.4 ± 14.68	7.0 ± 1.16	1348.2 ± 447.80	81.7 ± 6.09	1083.3 ± 106.88
***P*-value**	.627	.078	.273	.518	.545	.722

Values are presented as means ± SD.

Abbreviations: HSD17B1-KI, 17β-hydroxysteroid dehydrogenase 1 Ser143Ala knock-in; WAT, white adipose tissue; WT, wild-type.

^*a*^Determined from samples in the proestrus or estrus phase.

### The Lack of HSD17B1 Activity Leads to Upregulation of Ovarian Steroidogenesis and Mild Hyperandrogenism

**Figure 4.**
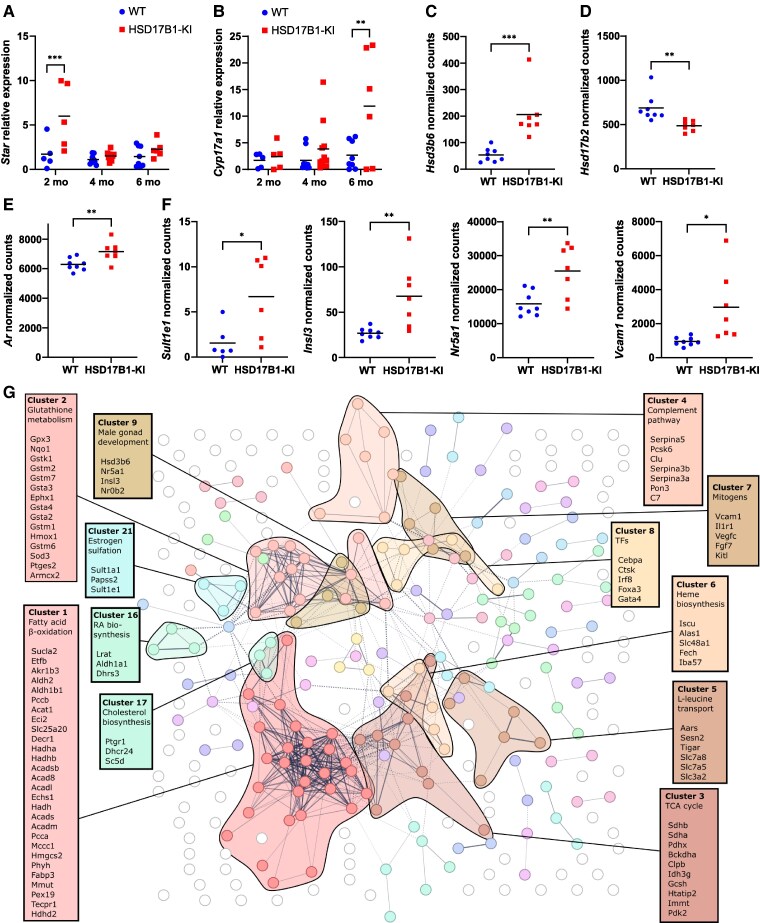
Ovarian gene expression was affected by HSD17B1 inactivation. (A) Relative ovarian expression of *Star* mRNA was increased in 2-month-old HSD17B1-KI females at the estrus stage of the cycle (n = 5) but not at 4 (WT n = 9, HSD17B1/KI n = 11) or 6 months (WT n = 8, HSD17B1-KI n = 7). (B) An increasing trend was visible in HSD17B1-KI ovary *Cyp17a1* expression in the same animals and was significant at 6 months. (C) mRNA sequencing revealed an upregulation of *Hsd3b6* in 6-month-old HSD17B1-KI, whereas (D) *Hsd17b2* was downregulated. (E) *Ar*, as well as (F) androgen-responsive genes *Sult1e1*, *Insl3*, *Nr5a1*, and *Vcam1,* were also observed to be upregulated in mRNA sequencing data. (G) STRING interaction analysis and clustering of the differentially expressed genes in 6-month-old females further revealed several clusters of interest upregulated in HSD17B1-KI. In (A-F), data are presented as individual values, with lines indicating the means. *P < .05, **P < .01, ***P < .001. Abbreviations: HSD17B1, 17β-hydroxysteroid dehydrogenase 1; HSD17B1-KI, 17β-hydroxysteroid dehydrogenase 1 Ser143Ala knock-in; WT, wild-type.

Due to the changes observed in steroid concentrations, we analyzed the mRNA expression of key rate-limiting components of steroidogenesis in WT and HSD17B1-KI ovaries at several time points. The levels of the transcripts of steroidogenic acute regulatory protein *Star* and the steroidogenic enzyme *Cyp17a1* remained similar between WT and HSD17B1-KI females when analyzed globally. However, when analyzing the subsets of animals in the estrus cycle phase, a trend for higher *Star* and *Cyp17a1* expression was observed in HSD17B1-KI ovaries. The difference reached statistical significance in 2-month-old mice for *Star* [fold change (FC) = 3.5, *P* ≤ .001] and 6-month-old mice for *Cyp17a1* (FC = 4.4, *P* = .003) (Fig. 4A and 4B).

To further understand the potentially altered gene expression in the HSD17B1-KI ovaries, we performed a global transcriptome analysis of the ovaries at 6 months of age and during the estrus phase (35). The analysis identified 456 differentially expressed (DE) genes between WT and HSD17B1-KI, with the majority of these (314, ie, 69%) being upregulated. All DE genes are presented in Table S1 (34). Besides *Cyp17a1,* out of known enzymes involved in steroidogenesis, only *Hsd3b6* was significantly upregulated (FC = 3.9, *P* = .0008; Fig. 4C), although the transcript count in HSD17B1-KI was several thousand-fold lower (205 ± 88 vs 384 000 ± 36 000) than that observed for *Hsd3b1*. Concurrently, the estrogen-inactivating enzyme *Hsd17b2* was marginally downregulated (FC = 0.7, *P* = .0067; Fig. 4D). No significant differences were observed in canonical enzymes of steroidogenesis, such as *Cyp11a1* and *Hsd3b1,* or other HSD17B enzymes. Other genes of interest identified in the data included the slightly upregulated androgen receptor *Ar* (FC = 1.14, *P* = .0097; Fig. 4E), as well as a clear upregulation of several genes known to be markers of androgen-linked Leydig cell maturation in male gonads. Those included sulfotransferase *Sult1e1* (FC = 5.0, *P* = .0270), insulin-like *Insl3* (FC = 2.5, *P* = .0066), steroidogenic factor *Nr5a1* (FC = 1.6, *P* = .0066), and vascular cell adhesion protein *Vcam1* (FC = 3.1, *P* = .0172; Fig. 4F).

Furthermore, STRING protein interaction analysis of the upregulated transcripts revealed several major clusters and their associated gene ontology terms [Fig. 4G; Table S2 (34)]. The largest cluster, cluster 1, comprised 28 transcripts of highly interconnected proteins associated with the degradation of valine, leucine, and isoleucine, as well as mitochondrial fatty acid β-oxidation. Cluster 2 included 15 transcripts of proteins associated with glutathione metabolism, cluster 3 those associated with the oxidoreductase complex and citric acid cycle (10 transcripts), and cluster 4 those associated with the serine protease inhibition and the complement pathway (7 transcripts). Cluster 5 included 6 transcripts coding for proteins associated with L-leucine transport, and cluster 6 included 5 genes associated with heme biosynthesis. Cluster 7 comprised 5 mitogens and signaling molecules and was closely associated with cluster 8, which included 5 transcription factors. Finally, cluster 9 included 4 transcripts coding for proteins associated with male gonad development. A high level of interconnectivity was also observed between the genes in clusters 1, 3, and 6 on the one hand and between clusters 2, 7, 8, and 9 on the other (Fig. 4G). In addition, several smaller clusters of 3 or 2 genes were also identified in the data. Of these, the most interesting for the ovarian phenotype and steroidogenesis were clusters 16 (retinoic acid biosynthesis), 17 (cholesterol biosynthesis), and 21 (estrogen sulfation). However, many of the top 10 most upregulated genes were not included in any of the major clusters, including calcium-sensing receptor *Casr* (FC = 11.8, *P* = 1,6E-5), aquaporin *Aqp2* (fc = 7.2, *P* = .0002), ATPase subunit *Atp12a* (FC = 6.0, *P* = .003), calcium-dependent secretion activator *Cadps* (FC = 5.8, *P* = .016), and calbindin *Calb2* (FC = 5.7, *P* = .016) (34).

The 142 downregulated genes, on the other hand, did not cluster as extensively in the STRING analysis, nor did they significantly affect the clustering when analyzing all DE genes vs only the upregulated genes. Identified downregulated clusters and their gene ontology terms included cell adhesion (5 transcripts), histidine catabolism (4 transcripts), amino acid transport (4 transcripts), and triglyceride biosynthesis (4 transcripts).

### HSD17B1 Inactivation Does Not Affect Bone Parameters in Female Mice

**Figure 5.**
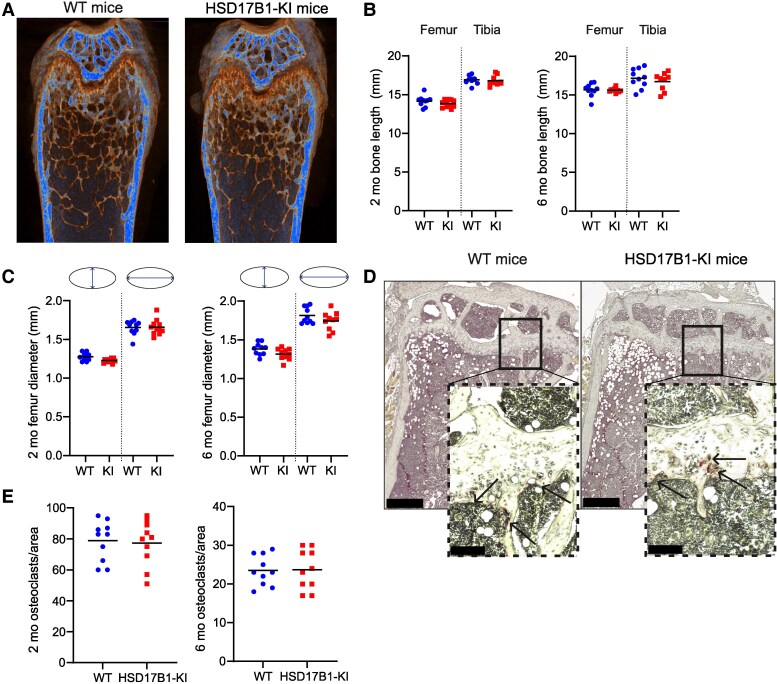
Bone parameters were unchanged in females with HSD17B1 inactivation. (A) Representative reconstructed micro-computed tomography images of bone microstructure in WT and HSD17B1-KI femur. Blue represents the highest tissue mineral density. (B) Femur and tibia length did not differ between WT and HSD17B1-KI females at 2 (WT n = 10, HSD17B1-KI n = 11) or 6 months of age (WT n = 10, HSD17B1-KI n = 10). (C) No differences were observed in femur diameter (sagittal or coronal) at 2 months or 6 months either. (D) Representative images of TRAcP staining of the tibia of WT and HSD17B1-KI females at the age of 6 months, with arrows indicating multinucleated TRAcP-positive osteoclasts below the growth plate, with (E) showing the similar number of osteoclasts counted in histological sections at 2 months or 6 months in both WT and HSD17B1-KI females. In (E), scale bars in wider field pictures represent 1000 µm and in closer pictures 200 µm. In (B, C, E), data are presented as individual values, with lines indicating the means. *P  *<* .05, **P < .01, ***P < .001. Abbreviations: HSD17B1, 17β-hydroxysteroid dehydrogenase 1; HSD17B1-KI, 17β-hydroxysteroid dehydrogenase 1 Ser143Ala knock-in; TRAcP, tartrate-resistant acid phosphatase; WT, wild-type.

As bone is a well-known estrogen target tissue, we analyzed various bone parameters of WT and HSD17B1-KI females to define the potential long-term effects of HSD17B1 inactivation on estrogen action in this peripheral target tissue in mice. The femur and tibia length and sagittal and coronal cross-sectional diameters of the femur were similar between WT and HSD17B1-KI mice at 2 or 6 months of age (Fig. 5A-5C). HSD17B1 inactivation did not alter either the trabecular or cortical bone parameters at either 2 or 6 months of age (Tables 4 and 5), and histological analysis revealed no differences in osteoclast number between WT and HSD17B1-KI female mice (Fig. 5D and 5E). Based on these data, bone quality or quantity was not significantly affected by HSD17B1 inactivation, which is in line with the observed normal concentrations of circulating E2 in HSD17B1-KI females.

**Table 4.** Trabecular bone parameters in the femurs of 2- and 6-month-old WT and HSD17B1-KI females

	Tissue volume (mm^3^)	Bone volume (mm^3^)	Bone/tissue volume (%)	Trabecular thickness (mm)	Trabecular separation (mm)	Trabecular number (mm^−1^)
**2 months**						
**WT**	2.40 ± 0.08	0.79 ± 0.06	32.86 ± 2.29	0.08 ± 0.01	0.19 ± 0.02	4.26 ± 0.44
**HSD17B1-KI**	2.38 ± 0.13	0.80 ± 0.09	33.65 ± 2.61	0.08 ± 0.01	0.19 ± 0.02	4.09 ± 0.42
**6 months**						
**WT**	1.93 ± 0.1	0.69 ± 0.08	35.67 ± 3.29	0.11 ± 0.01	0.18 ± 0.03	3.35 ± 0.54
**HSD17B1-KI**	1.88 ± 0.12	0.65 ± 0.04	34.73 ± 1.75	0.11 ± 0.01	0.18 ± 0.02	3.27 ± 0.21

Values are presented as means ± SD.

Abbreviations: HSD17B1-KI, 17β-hydroxysteroid dehydrogenase 1 Ser143Ala knock-in; WT, wild-type.

**Table 5.** Cortical bone parameters in the femurs of 2- and 6-month-old WT and HSD17B1-KI females

	Tissue volume (mm^3^)	Bone volume (mm^3^)	Bone/tissue volume (%)	Mean total cortical tissue area (mm^2^)	Mean total cortical bone area (mm^2^)	Endocortical area (mm^2^)	Mean total cortical bone perimeter (mm)	Cortical thickness (mm)
**2 months**								
**WT**	0.43 ± 0.01	0.16 ± 0.01	36.74 ± 2.26	1.65 ± 0.07	0.61 ± 0.05	1.05 ± 0.05	9.21 ± 0.34	0.13 ± 0.01
**HSD17B1-KI**	0.42 ± 0.03	0.15 ± 0.01	37.07 ± 1.85	1.61 ± 0.11	0.60 ± 0.06	1.02 ± 0.06	8.99 ± 0.36	0.13 ± 0.01
**6 months**								
**WT**	0.44 ± 0.03	0.23 ± 0.01	51.87 ± 1.84	1.71 ± 0.12	0.89 ± 0.05	0.82 ± 0.08	9.4 ± 0.47	0.19 ± 0.01
**HSD17B1-KI**	0.42 ± 0.03	0.22 ± 0.01	51.82 ± 1.94	1.65 ± 0.10	0.85 ± 0.04	0.79 ± 0.07	9.27 ± 0.51	0.18 ± 0.01

Values are presented as means ± SD.

Abbreviations: HSD17B1-KI, 17β-hydroxysteroid dehydrogenase 1 Ser143Ala knock-in; WT, wild-type.

To further evaluate the potential impact of local HSD17B1 activity and its inhibition on estrogen balance in bone, we conducted experiments using cultured human osteoclasts, primary osteoblasts, and osteoblasts derived from MSCs. Mature osteoclasts were seen to partially convert E1 in media to an unknown metabolite, which was not reflected in E2 concentration, and to partially inactivate E2 to E1, demonstrating only oxidative HSD17B activity, presented in Fig. S4A (34). In cultured primary osteoblasts and MSC-derived osteoblasts, again no conversion of E1 to E2 was observed, but rather a modest conversion of E2 to E1 was apparent in primary osteoblasts, and E1 was again seen to be converted to an unknown metabolite in MSC-derived osteoblasts (Fig. S4B and S4C) (34). Notably, the addition of an HSD17B1 inhibitor did not affect the observed E1 and E2 concentrations, further demonstrating that human osteoclasts and osteoblasts do not possess HSD17B1 activity in vitro (Fig. S4A-S4C) (34).

## Discussion

The Ser143Ala point mutation has been shown to abolish human and mouse HSD17B1 activity in several previous studies (17, 36). Molecular modeling analysis at the protein 3D structure level indicated that the mutation disrupts the widely conserved short-chain dehydrogenases/reductases catalytic triad, Ser-Tyr-Lys, which facilitates the reduction of E1 to E2 at the C17 position of the steroidal ring (18). However, compared to the human enzyme, mouse HSD17B1 is less specific for the estrogens, and our current analysis revealed the most significant difference between the mouse and human enzyme structures, allowing the mouse enzyme to use both A4 and E1 as substrates. Leu150 residue in the binding pocket has been previously identified as being responsible for the androgen/estrogen discrimination of human HSD17B1 (22), but this residue is also present in the mouse enzyme. Based on our analysis, the presence of His222 interacting with the C17 hydroxyl group is instead a major factor enabling androgens to bind the human enzyme in reverse mode. This has been previously suggested to contribute to DHT inactivation by human HSD17B1 under certain conditions (22, 37). However, the replacement of His222 with glycine in the mouse HSD17B1 allows androgens to bind in a proper orientation for the catalysis. Moreover, E1 has been suggested to inhibit human HSD17B1 by adopting a reverse binding mode when excess substrate is available (38). This type of substrate-induced inhibition or dead-end complex formation is unlikely to exist in the mouse HSD17B1, because the C17-hydroxyl cannot acquire a similar coordination without the presence of His222.

We have previously demonstrated that the Ser143Ala mutation disrupts A4 to T conversion by mouse HSD17B1 (17). In line with this, we now confirmed that the point mutation inactivates the E1 to E2 conversion in cultured cells, and the intraovarian and serum steroid concentrations were altered due to the impaired HSD17B activity. The concentrations of precursor steroids E1 and A4 were increased in HSD17B1-KI female ovaries, while the concentrations of active sex steroids, E2 and T, remained unchanged. This indicates that the increased precursor concentrations effectively compensate for the absence of HSD17B1. We have recently reported a similar increase in the amount of the precursor steroid in the testes of HSD17B3 knockout mice (39). The higher levels of DHT, but not T, in the HSD17B1-KI ovaries suggest that DHT is produced from excess precursor androgens through the so-called backdoor pathway, without T serving as an intermediate (40). Interestingly, the serum concentration of T was increased in HSD17B1-KI females, likely due to increased circulating A4 being activated into T in peripheral tissues. A similar elevation of circulating, but not testicular, T, alongside high A4, has also been previously observed in male mice lacking HSD17B3 (39).

Reflecting the normal ovarian E2 production and its appropriate levels in circulation, the observed effects in the females were minimal, and the inactivation of the enzyme did not disturb female development or puberty onset. The estrous cycle of the HSD17B1-KI mice progressed mainly normally, although some animals spent more time in the metestrus phase, and the HSD17B1-KI females were fertile. While some of them presented with an increased time between litters in breeding experiments, others produced a litter in a completely normal manner. Overall, the inactivation of HSD17B1 appeared to have a variable, yet mild, effect on female reproduction.

Despite the slightly lower body weight of young HSD17B1-KI females, the hormonal disruption did not affect the weights of reproductive or other tissues, and the difference in body weight did not persist into adulthood, leaving the cause of the initial lower body weight unclear. However, even though a reduced ovary weight was observed in old HSD17B1-KI females, histology did not reveal any obvious disruptions of development or the follicular cycle, suggesting that despite a slight intraovarian hormonal imbalance in the absence of HSD17B1, the mouse ovaries maintained their proper function. The reduced ovary size at old age did not reflect any insufficiency, also evidenced by the normal response to superovulation.

The stimulation of ovarian steroidogenesis in HSD17B1-KI was still under the control of the hypothalamus-pituitary-gonadal (HPG) axis, as evidenced by the increase in circulating LH. Interestingly, *Cyp17a1* mRNA seemed to be consistently and increasingly upregulated in successive age cohorts. In contrast, the preceding components of steroidogenesis, *Star*, and *Cyp11a1*, were not, although all are shown to be under direct LH regulation (41). However, this is in accordance with our previous data on a model where the entire genomic region of mouse HSD17B1 was deleted and replaced with a Lac-Z reporter gene. In those mice, *Star* and *Cyp11a1* were upregulated in the proestrus but not in the estrus phase, and vice versa for *Cyp17a1* (14). The differential upregulation was also reflected in the steroid concentrations, as A4 and E1 were increased, but the precursor progesterone, the substrate for CYP17A1, was not. Notably, LH remained elevated, although intraovarian and circulating sex steroids reached normal or above normal concentrations, indicating that persistent HPG upregulation is necessary for compensation. A similar phenomenon has been reported in the androgen production of male mice lacking HSD17B3, where LH remained high despite high serum T, but was normalized by the reintroduction of functional HSD17B3 (39, 42). The interstitial hyperplasia and hypertrophy observed in some HSD17B1-KI ovaries are also indicative of the prolonged and increased LH exposure (43).

The compensatory mechanism of E2 production in the absence of HSD17B1 activity remains unclear, as no upregulation of any enzyme known to catalyze the same reaction was detected. It is likely that the upregulation of the earlier rate-limiting steps of steroidogenesis and the increase of precursor steroids are enough to allow other enzymes with less efficient reductive HSD17B activity to produce significant amounts of E2. At least HSD17B7 and HSD17B12 are expressed in the ovaries and are capable of catalyzing this reaction to a certain degree (44, 45), but it is also possible that 1 or more enzymes with yet unidentified reductive HSD17B activity are involved. The downregulation of HSD17B2, converting E2 to E1, is potentially a response to the decreased E2/E1 ratio in the ovaries, although its regulation by estrogens is not well established. HSD3B6 is primarily known to be involved in testicular steroidogenesis of mice, in mature Leydig cells under LH control (46). However, the detected expression was far lower than that of HSD3B1, and thus, the role of its upregulation in the total ovarian HSD3B activity is most likely minimal.

Besides *Hsd3b6,* we noticed that the top DE transcripts identified in RNA sequencing also included several other markers associated with fully mature adult-type Leydig cells, including *Sult1e1*, *Insl3*, *Nr5a1*, and *Vcam1* (46, 47). Some of the upregulation could be a response to LH stimulation in theca (*Insl3*, *Nr5a1*) or granulosa (*Sult1e1*) cells (48-50). However, upregulation of these genes in the ovaries has also been reported following DHT exposure in mice or has been associated with hyperandrogenism in human polycystic ovary syndrome (PCOS) (51-53). This, together with the observed upregulation of the androgen receptor itself, suggests an increased androgen exposure in the HSD17B1-KI ovaries (54).

STRING analysis revealed several large clusters of DE genes involved in mitochondrial metabolism, including wide upregulation of enzymes involved in fatty acid and other CoA-conjugate oxidation (cluster 1), TCA cycle (cluster 3), transporters of L-leucine and other amino acids (cluster 5), and heme biosynthesis (cluster 6). LH has been shown to increase mitochondrial glucose, lipid, and amino acid metabolism in luteal cells, and the TCA cycle and fatty acid oxidation are crucial for steroidogenesis and follicle maturation (55, 56). Acyl- and acetyl-CoA metabolism can also provide precursors for cholesterol, and some upregulation was also observed in other components of cholesterol biosynthesis (cluster 17), potentially providing more substrate for local steroidogenesis.

The second-largest cluster of genes (cluster 2) was related to glutathione metabolism, including a large number of glutathione S-transferase isoenzymes. They are mainly known as detoxifying enzymes, but several types of human glutathione S-transferase have been identified primarily in steroidogenic cells and catalyze steroid Δ5-4 isomerization, complementing HSD3B enzyme activity (57, 58), and in line with this, the expression of some of the isoenzymes has been shown to be regulated by gonadotropins in bovine ovary (59).

Clusters 7, 8, and 9 included many factors reported to be upregulated in PCOS women or PCOS models, induced either by excess LH stimulation, androgens, or yet undetermined causes (60). These include the aforementioned *Insl3*, *Nr5a1*, and *Vcam1* but include also *Cebpa, Foxa3*, *Gata4*, and *Vegfc* (60-63). Additionally, many of the highly upregulated genes were also associated with steroidogenesis or sex hormone action, including the most upregulated gene *Casr*, which has been identified as a marker of hyperandrogenic PCOS (64).

Finally, a small cluster of genes associated with retinoic acid (RA) biosynthesis was found to be upregulated. RA biosynthesis and signaling are crucial for folliculogenesis, as well as ovarian steroidogenesis (65). In particular, ALDH1A1, which was highly expressed and significantly upregulated in HSD17B1-KI ovaries, has been identified as enhancing both granulosa cell proliferation and ovarian steroidogenesis in sows (66). Interestingly, HSD17B2, found to be downregulated in HSD17B1-KI ovaries, has previously been linked to RA metabolism in our previous study with HSD17B2 overexpressing mice (67).

The reproductive phenotype in HSD17B1-KI females was significantly milder than in female mice with a replacement of the entire *Hsd17b1* gene with a LacZ insertion (14). Although females in that model developed normally, and steroid concentrations and ovary steroidogenic gene expression were very similar to those in the present study, they exhibited significantly poorer fertility and more drastic ovarian interstitial hypertrophy/hyperplasia. This reproductive phenotype was likely affected by extreme off-target effects due to the reported silencing of the neighboring gene *Naglu* (15). Therefore, we conclude that the inactivating point mutation presented here models the consequences of pharmacological inhibition of HSD17B1 activity more accurately. The present data revealed that the absence of HSD17B1 activity in mouse ovaries was compensated for by upregulation of upstream steroidogenic steps, mediated by the HPG axis. This resulted in normal E2 concentration via a noncanonical synthesis pathway, with no significant disruption to reproductive health.

However, the increased LH stimulation and accumulation of A4 and DHT in the mutant ovaries led to a PCOS-like phenotype with interstitial hyperplasia (68) and altered gene expression. In line with our data in mice, lowered expression of HSD17B1 has been reported in the blood of PCOS patients (69), and a case report associated a homozygous splice variant with poor ovarian response and ovarian cysts (70). Furthermore, HSD17B1 expression was shown to be lowered in granulosa luteal cells of women with PCOS (71). Thus, the ovarian phenotype in the HSD17B mutant mice provides further evidence that reduced ovarian HSD17B1 activity may predispose to PCOS, and the HSD17B1KI mice present a novel animal model to identify the mechanisms behind the hyperandrogenism of PCOS.

Other aspects of the phenotype, particularly the estrogen-sensitive bone tissue, were not affected by the lack of HSD17B1 activity, likely due to normal circulating E2 levels. Similarly, available data indicate that pharmacological inhibition of HSD17B1 activity in premenopausal women does not alter circulating E2 levels (72), although intraovarian steroid concentrations remain undetermined. Combined with our current findings showing that human osteoclasts and osteoblasts do not exhibit detectable reductive HSD17B activity, the data suggest that HSD17B1 inhibition is unlikely to disrupt estrogen balance in bone tissue. Thus, even the complete blockade of HSD17B1 activity was observed to have little impact on female mouse health in general, due to effective compensatory mechanisms of sex steroid biosynthesis.

## Data Availability

The RNA sequencing data have been deposited in the GEO database under accession number GSE306070 (35). All the other data are presented in the manuscript or in the supplementary materials. Additional requests can be directed to the corresponding author.
